# Successful treatment of vancomycin-resistant enterococcal infection of an external ventricular drain with 2 weeks of intravenous linezolid

**DOI:** 10.1099/acmi.0.000335

**Published:** 2022-03-29

**Authors:** Michael D. Cearns, Bruce T. McLintock, Nigel Suttner, Kamaljit Khalsa

**Affiliations:** ^1^​ Department of Neurosurgery, Institute of Neurological Sciences, 1345 Govan Road, Glasgow G51 4TF, UK; ^2^​ Department of Medical Microbiology, Queen Elizabeth University Hospital, 1345 Govan Road, Glasgow G51 4TF, UK

**Keywords:** hydrocephalus, external ventricular drain, vancomycin-resistant enterococcus, linezolid

## Abstract

Hydrocephalus is a common condition worldwide, and is frequently managed by diversion of cerebrospinal fluid (CSF), either externally with a drain or internally with a shunt. An external ventricular drain (EVD) can be an essential treatment modality, but is associated with a risk of infection, most commonly caused by *Staphylococcal* species, which can result in meningitis or ventriculitis and a delay in the definitive management of the hydrocephalus. Here, we report the case of a patient who required an EVD to manage post-operative hydrocephalus following a craniotomy and microvascular decompression for trigeminal neuralgia. He subsequently developed EVD-associated infection with a vancomycin-resistant *Enterococcus faecium* (VRE), which was treated successfully with a 2-week course of intravenous linezolid monotherapy. The authors believe this to be the only described case of successful treatment within this time frame of a CSF VRE infection associated with indwelling foreign material.

## Introduction

Hydrocephalus is defined as an abnormal accumulation of cerebrospinal fluid (CSF) within the ventricles of the brain, caused either by subnormal reabsorption or, rarely, overproduction of CSF [1]. It can lead to ventricular dilatation, raised intracranial pressure and death, and whilst associated with various conditions, it has long been recognized as a complication of neurosurgical intervention to the posterior cranial fossa [2]. Whilst a reliable global estimation of incidence is unavailable [3], hydrocephalus is one of the most common brain disorders and leads to 70 000 hospital admissions per year in the USA alone [4]. Treatment of hydrocephalus typically involves diversion of CSF, either internally via a shunt or externally via a drain. An external drain is a temporary measure, with the proximal end placed either into a cerebral ventricle (external ventricular drain, EVD) or the lumbar subarachnoid space (lumbar drain), with the distal end draining into a collecting system. CSF shunts and drains are both associated with infection, and whilst they are recognized as essential treatment modalities, it is important to balance their benefits against these infection-related complications [5].

The rate of EVD-associated infection varies from 0–36 % [6], although most case series report rates of 5–10 % [7, 8]. Whilst the risk factors for EVD-associated infection remain poorly understood [9], there are several factors that appear to increase the risk of its development, including duration of CSF drainage, co-existing systemic infection and previous neurosurgical intervention. Whilst there is some controversy regarding any association between duration of CSF drainage and risk of infection, there is a consensus that a period of more than 5 days is likely to be significant [8].

The causative organisms of shunt- and drain-associated infections are most commonly those that colonize the scalp and skin of the back, and increasingly those acquired in a healthcare environment. Whilst *Staphylococcal* infection remains the most common cause (55–90 % incidence) [10], Gram-negative bacilli, Gram-positive bacilli, fungi and antimicrobial-resistant bacteria have also been documented.

In this report, we review the case of a 67-year-old gentleman with an EVD-associated infection caused by vancomycin-resistant *Enterococcus faecium* (VRE).

## Case presentation

### History and examination

A 67-year-old right-handed male was referred with an 8-year history of classical trigeminal neuralgia affecting the distribution of the mandibular division of the trigeminal nerve. He had a background of hypertension and a previous stroke, for which he took clopidogrel. He described paroxysmal ‘electric’ pain, occurring spontaneously or brought on by touching, eating or talking. This had initially responded to carbamazepine before becoming more frequent and severe despite medical therapy. On examination, the patient’s Glasgow coma score was 15/15. There was a left lower limb weakness of MRC grade 4/5. Sensory examination was normal, there were no cranial nerve deficits and he had clear speech. General systemic examination was normal.

### Imaging

**Fig. 1.**
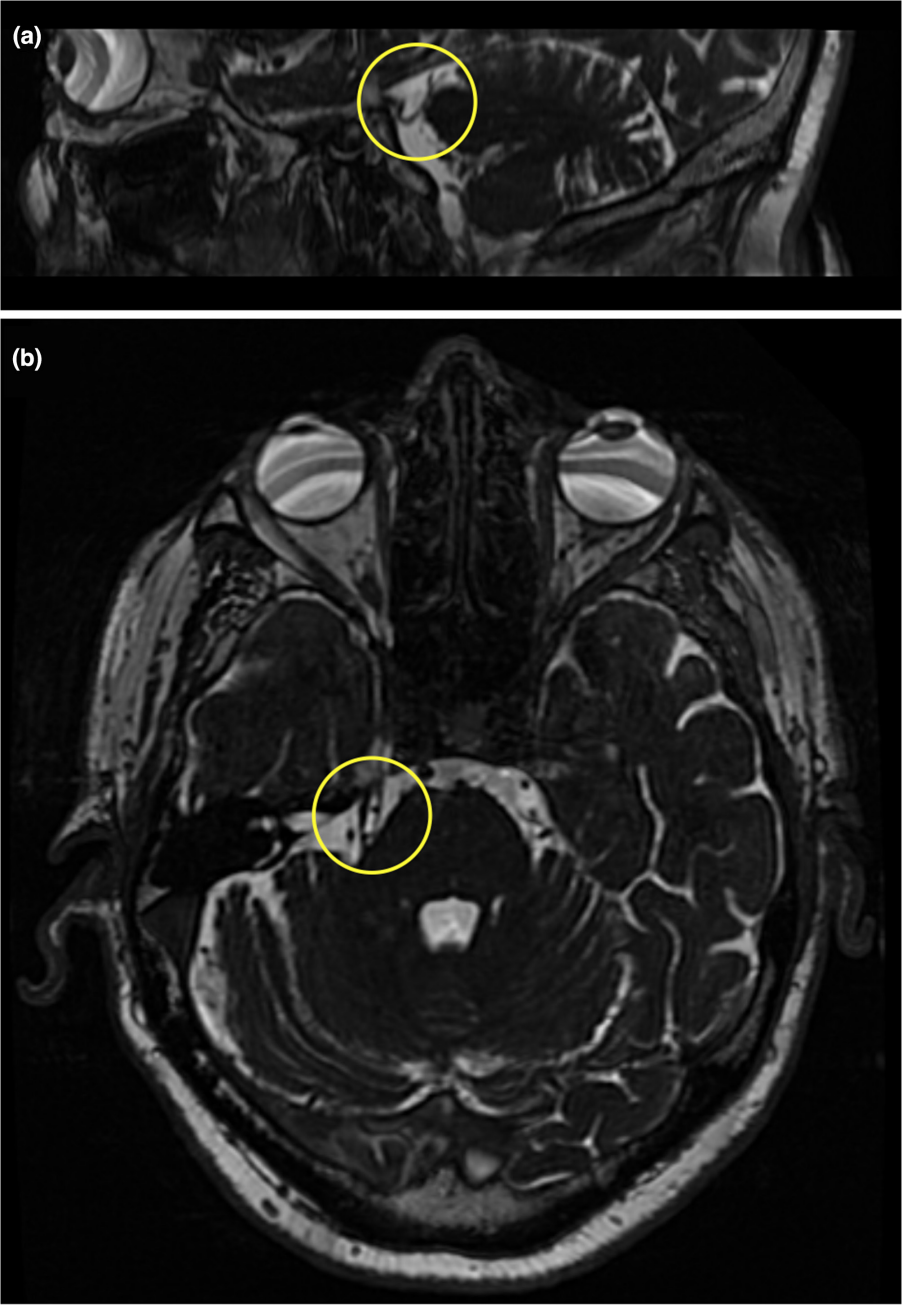
Sagittal (**a**) and axial (**b**) T2-weighted magnetic resonance images demonstrating neurovascular conflict on the right side (yellow circle) between the trigeminal nerve and a descending loop of the superior cerebellar artery.

Cerebral MRI and MR angiography revealed a descending loop of the superior cerebellar artery causing a neurovascular conflict with the trigeminal nerve on its entry into the pons (Fig. 1).

### Operation

The patient underwent a microvascular decompression of the right trigeminal nerve. This was performed via a right retro-auricular incision and a retrosigmoid craniotomy. Following dural and arachnoid opening, CSF was released, the cerebellum gently retracted along the venous sinus angle, and a polytetrafluoroethylene (PTFE) felt was placed between the superior cerebellar artery and the trigeminal nerve. The dura was tacked closed and covered with a pericranial graft and dural sealant, which was followed by layered closure of the galea aponeurotica and the skin.

### Post-operative course

**Fig. 2.**
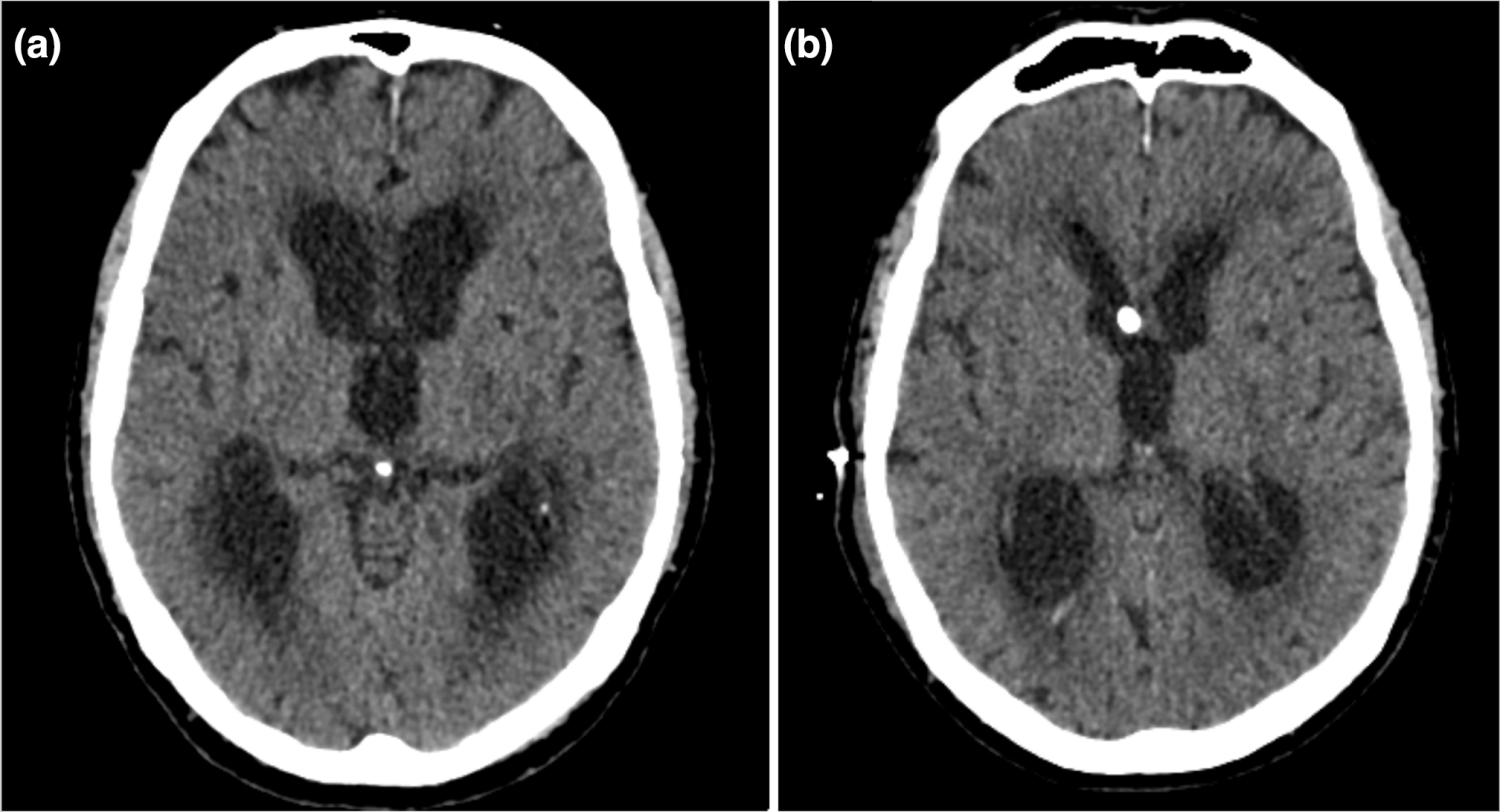
Axial non-contrast computed tomography scans taken (**a**) 8 weeks post-operatively, showing hydrocephalus, and (**b**) following insertion of an external ventricular drain into the right lateral ventricle, enabling decompression of the ventricular system.

The patient’s early post-operative course was marked by a period of hypertension as well as delirium attributed to pneumonia. This resolved following empirical treatment with intravenous (IV) amoxicillin and he was discharged home 7 days post-operatively, having had no further episodes of facial pain. However, he re-presented 8 weeks post-operatively with new confusion, and a CT head revealed hydrocephalus (Fig. 2a). He underwent lumbar puncture at this stage, which demonstrated no evidence of CSF infection. He underwent operative exploration by reopening of the original incision. At operation the cerebellum was stuck to the overlying dura, and the path towards and surrounding the trigeminal nerve was covered with a thickened layer of arachnoid, sealing off all the CSF cisterns. It was felt that this was likely to be a reaction to the PTFE felt used to separate the nerve from the artery, and the cisterns were reopened as much as safely possible. A lumbar drain was left *in situ*, which was removed 4 days post-operatively following an improvement in his confusion.

Two days later, the patient became agitated, pyrexial and tachycardic, with signs of meningism, and lumbar puncture demonstrated a raised opening pressure of 32 cmH20 and a white cell count of 2230, with a 90 % predominance of polymorphs. He was therefore started empirically on IV ceftriaxone and vancomycin. *Staphylococcus epidermidis* was subsequently identified from the CSF sample from enrichment culture only, which was felt to be a contaminant.

**Fig. 3.**
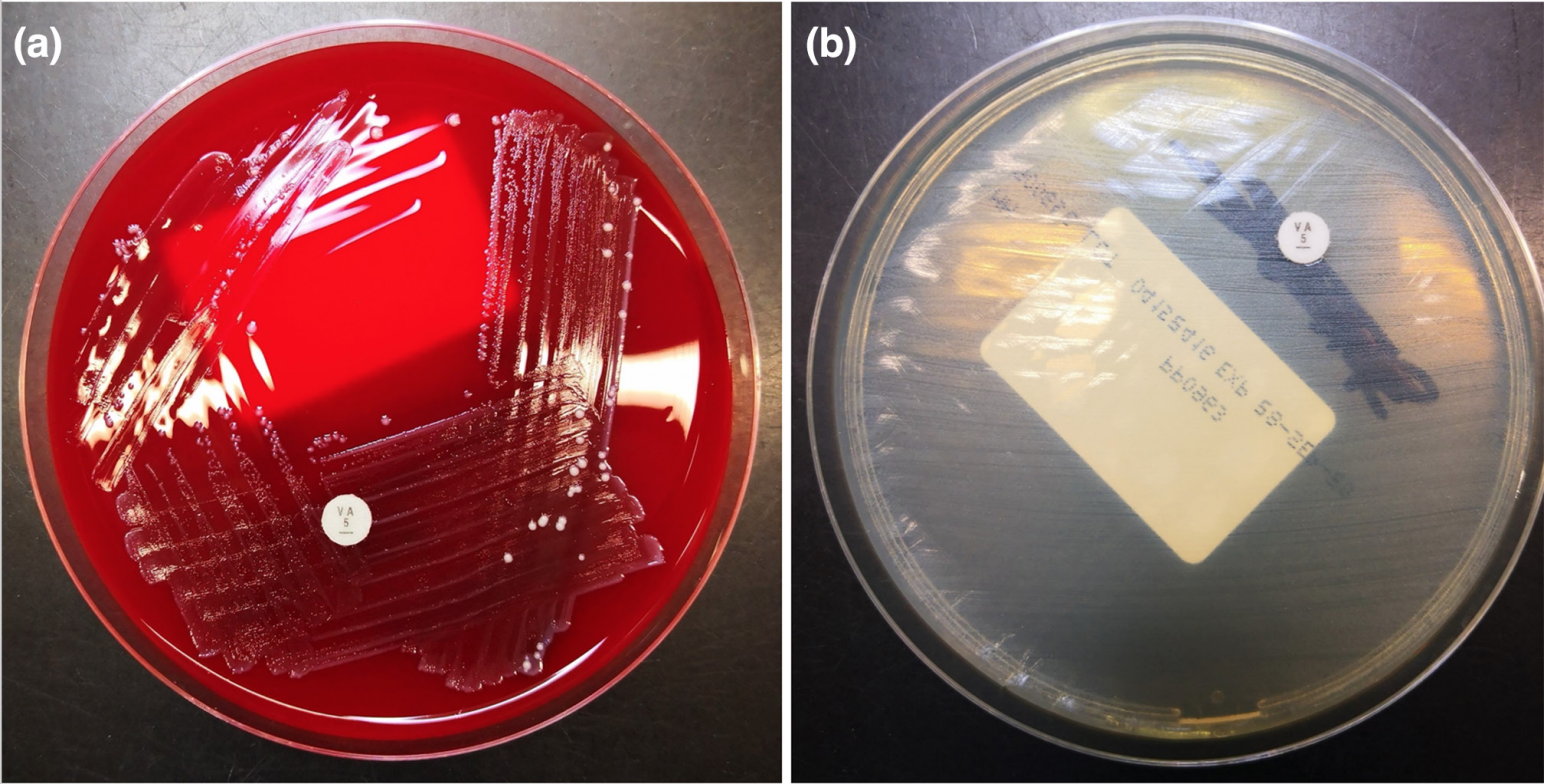
Vancomycin-resistant *E. faecium* growing up to and around a vancomycin disc on both Columbia blood agar (**a**) and Mueller–Hinton (**b**) agar plates.

The following day his conscious level deteriorated and he underwent insertion of an external ventricular drain (Fig. 2b); at operation there was found to be turbid CSF under pressure. Despite 72 h of IV vancomycin and ceftriaxone, the patient continued to have ongoing episodes of both pyrexia and tachycardia. After discussion with the microbiology department, the antibiotics were changed to IV vancomycin and meropenem. A new EVD was inserted 10 days later, as the original was draining poorly. CSF cultures from the original EVD, taken prior to removal, were positive for *E. faecium *, identified through use of matrix-assisted laser desorption/ionization time-of-flight mass spectrometry (MALDI-TOF MS). Provisional sensitivity testing was suggestive of vancomycin resistance (Fig. 3) and subsequent sensitivity testing, as per the European Committee on Antimicrobial Susceptibility Testing (EUCAST) breakpoints, demonstrated that the organism was sensitive to linezolid [mean inhibitory concentration (MIC)=2 mg l^−1^) but resistant to amoxicillin (MIC>8 mg l^−1^), vancomycin (MIC>256 mg l^−1^) and teicoplanin (MIC>32 mg l^−1^). The organism also demonstrated high-level gentamicin resistance (MIC>512 mg l^−1^). Daptomycin MIC was 4 mg l^−1^ and, whilst there are no EUCAST breakpoints, this is sensitive according to the Clinical Laboratory and Standards Institute (CLSI) breakpoints, provided a dosing regime of 8–12 mg kg^−1^ per 24 h is used.

After further liaison with the microbiology department, vancomycin was therefore switched to a 2-week course of IV linezolid 600 mg every 12 h, with meropenem being discontinued after 2 weeks of therapy. Blood results were monitored regularly throughout the course of linezolid, but no change in dosing or frequency was indicated. After 2 weeks of linezolid, and multiple negative CSF cultures from the EVD, the infection was felt to have been treated successfully. The EVD was removed and the patient underwent insertion of a left-sided parietal ventriculoperitoneal shunt using a Codman programmable valve. Six weeks following hospital discharge he was alert, orientated, walking independently with a stick, and free of all neuralgia medications, with no ongoing facial pain.

Following discharge from hospital, the VRE sample was sent to Public Health England’s antimicrobial resistance and healthcare-associated infections (AMRHAI) reference unit. They performed susceptibility testing on the sample and, based on the pattern of MICs, determined it to be a VanA type VRE.

## Discussion

In this paper we report the case of an EVD-associated infection with vancomycin-resistant *E. faecium *. *Enterococci* are predominantly environmental organisms found most commonly in soil, water and the gastrointestinal (GI) tract of animals [11]. They are facultatively anaerobic Gram-positive cocci, seen as anything from single cells to very long chains. *Enterococci* are able to grow in extreme conditions; at a pH of up to 9.6, in 6.5 % sodium chloride (NaCl) agar, and at temperatures ranging from 10–45 °C. In addition, arabinose-containing agar plates can be used to differentiate *E. faecium* from other *Enterococci* due to its ability to ferment arabinose.

*Enterococci* are a leading and increasing cause of nosocomial infection due to the ease of transmission between both patients and institutions. In 2017 in England, Wales and Northern Ireland, the overall incidence of enterococcal bacteraemia was 13.1 per 100 000 population, an increase from 9.9 in 2010 [12]. The rate of resistant enterococcal infections is also increasing, with 30 % caused by VRE, 90 % of which are *E. faecium.* enterococcal vancomycin resistance may be inherited (*Enterococcus casseliflavus, Enterococcus gallinarum*) or acquired (*Enterococcus faecalis, E. faecium*). Six different resistance mechanisms have been named on the basis of their ligase genes – VanA–E and VanG – all of which result in the production of a peptidoglycan with decreased affinity to the glycopeptides [11].

As well as being present in the environment, *Enterococci* are well-adapted to live in the GI tract of humans. Whilst they are a minor population when compared to commensal anaerobes, they have a symbiotic relationship with these bacteria and with the immune system itself. Due to their inherent resistance to a number of antimicrobials, systemic antibiotic therapy has been found to alter the GI tract in favour of *Enterococci*. Use of broad-spectrum antibiotics with significant activity against anaerobes, but limited activity against *Enterococci*, favours the colonization of the GI tract by resistant E*nterococci* such as VRE.

There are many clinical manifestations of enterococcal infection, including urinary tract infections, bacteraemia, endocarditis, intra-abdominal and pelvic infections, skin and soft tissue infections and, rarely, meningitis. Cases of meningitis can be further subdivided into spontaneous and post-operative. Interestingly, whilst *Enterococci* account for 0.3–4 % of all cases of meningitis [13, 14], a single-centre retrospective study from Germany demonstrated that in EVD-associated infections, *Enterococcus* subspecies accounted for 19 % of cases [15], suggesting an increased proportion of enterococcal infections in the context of EVD placement. However, EVD-associated VRE infection appears to be seen less commonly, and a literature review by the authors of this report could only find a handful of previous cases [5, 16–18]. Patients with post-operative enterococcal meningitis usually present with an acute course of altered mental status, signs of meningeal irritation and fever, and analysis of CSF demonstrates pleocytosis, elevated protein and decreased glucose levels.

The Infectious Diseases Society of America (IDSA) guidelines on treatment of healthcare-associated ventriculitis/meningitis recommend treatment with vancomycin plus an anti-pseudomonal beta-lactam, in addition to strongly recommending the removal of an infected CSF drain if one is *in situ* [19]. In this case, the patient was first commenced empirically on vancomycin and ceftriaxone. However, as a result of further pyrexial episodes, antibiotics were escalated to vancomycin and meropenem. Again, there was no clinical improvement, and the patient’s EVD was changed at 10 days following insertion of the first EVD. Two days later, VRE was identified in CSF sampled from the first EVD prior to its removal, and at this point antibiotic therapy was changed to linezolid and meropenem. Interestingly, a 2-week course of systemic linezolid alone was able to provide bacterial clearance from the second EVD and intraventricular antibiotics were not necessary, unlike in several previous case reports, where antibiotics were required to be delivered by this route [5, 16, 17]. The potential use of chloramphenicol following 2 weeks of linezolid therapy was discussed; however, the patient was clinically stable after discontinuation of linezolid and no further antibiotics were prescribed.

In concordance with current evidence, our patient had several risk factors for EVD-associated infection, including prolonged duration of CSF drainage and previous neurosurgical intervention. Whilst the mechanism of infection remains unclear, our hypothesis in this case relates to vancomycin-resistant *E. faecium* detected in the stool sample of the patient following diagnosis of EVD-associated infection. We believe that the use of broad-spectrum ceftriaxone altered the GI environment to the benefit of enterococcal species, and the concurrent use of vancomycin led to selective pressures resulting in the colonization of the GI tract with VRE. This VRE subsequently led to an EVD-associated infection through retrograde spread from the distal end of the drain following contamination with VRE (the most common mechanism of meningitis/ventriculitis in those with CSF drains *in situ*) [10]. Haematogenous spread appears less plausible due to the absence of bacteraemia, and as there was no clinical evidence of wound infection, direct spread through the skin also appears less likely. Colonization of the drain at the time of surgery is difficult to exclude as a possible source. However, an initial culture of CSF from the EVD following insertion yielded no growth, and the subsequent presence of VRE in both the CSF from the EVD and in the stool led us to believe that retrograde infection was the most likely cause in this case.

## Conclusion

This patient was treated effectively with a 2-week course of IV linezolid, an oxazolidinone antibiotic that is bacteriostatic against *Enterococci* and has effective penetration into the CSF. A 2003 review of 39 cases of enterococcal meningitis found 15 attributable to VRE [14]. A further literature review found evidence of only seven cases treated effectively with IV linezolid [18]. Of these seven, only one patient was treated with a 2-week course, and this person did not have foreign material *in situ* at time of treatment. Therefore, our case report appears to be unique and suggests that it may be possible to treat EVD-associated VRE infection effectively with a 2-week course of systemic linezolid monotherapy. However, in addition to systemic linezolid, we would advocate removal of the infected EVD (in line with IDSA guidelines), with exchange of the EVD if indicated, as was the case with our patient. Further research and appropriate clinical trials would be required before any definitive recommendations could be made with regard to treatment of EVD-associated VRE infection.
